# A metabolomics perspective on 2 years of high-intensity training in horses

**DOI:** 10.1038/s41598-024-52188-z

**Published:** 2024-01-25

**Authors:** L. Johansson, S. Ringmark, J. Bergquist, E. Skiöldebrand, A. Jansson

**Affiliations:** 1 Department of Anatomy, Physiology and Biochemistry, Swedish University of Agricultural Sciences, P.O. Box 7011, 750 07 Uppsala, Sweden; 2 Department of Chemistry-BMC, Analytical Chemistry and Neurochemistry, Uppsala University, P.O. Box 599, 751 24 Uppsala, Sweden; 3 Department of Biomedical Sciences and Veterinary Public Health, Swedish University of Agricultural Sciences, P.O. Box 7028, 750 07 Uppsala, Sweden

**Keywords:** Biochemistry, Molecular biology, Physiology, Biomarkers

## Abstract

The plasma metabolomic profile of elite harness horses subjected to different training programmes was explored. All horses had the same training programme from 1.5 until 2 years of age and then high-intensity training was introduced, with horses divided into high and low training groups. Morning blood samples were collected at 1.5, 2, 2.5 and 3.5 years of age. The plasma was analysed using targeted absolute quantitative analysis and a combination of tandem mass spectrometry, flow-injection analysis and liquid chromatography. Differences between the two training groups were observed at 2 years of age, when 161 metabolites and sums and ratios were lower (e.g. ceramide and several triglycerides) and 51 were higher (e.g. aconitic acid, anserine, sum of PUFA cholesteryl esters and solely ketogenic AAs) in High compared with low horses. The metabolites aconitic acid, anserine, leucine, HArg synthesis and sum of solely ketogenic AAs increased over time, while beta alanine synthesis, ceramides and indole decreased. Therefore high-intensity training promoted adaptations linked to aerobic energy production and amino acid metabolism, and potentially also affected pH-buffering and vascular and insulin responses.

## Introduction

Metabolomics studies on the effects of sport and exercise in humans have been performed for decades, e.g. a bibliometric review published in 2022 located 122 relevant papers in databases using keywords associated with exercise and metabolomics^1^. However, the review showed that untargeted metabolomics studies and studies on short-term effects of exercise have dominated and that improvements in study design, method standardisation and data interpretation are needed to improve the quality of metabolomics studies. Lack of high-quality studies in this area was also highlighted in a study that identified a need for more cross-sectional and exercise training studies to improve understanding of the response to chronic exercise workloads^2^.

In the review referred to above^1^, metabolomics studies were classified into short-term (≤ 1 week) and long-term (> 1 week). From a practical exercise perspective, these periods are remarkably short and the results obtained in such studies may not accurately represent the effects achieved by the years of training commonly required to become an elite athlete, both in human sports and in equine athletes. Race and harness horses are elite athletes, but there is a lack of metabolomics studies of horses in training covering periods longer than 12 weeks^3–6^. Accordingly, there has been no longitudinal study on the metabolomics of horses subjected to true elite training, from the start of training until fit for racing. In addition, there have been no metabolomics studies to evaluate different training programmes.

The aims of this study were thus to: (1) explore differences in the metabolomic profile in plasma of young elite harness horses kept under standardised conditions and subjected to two different training programmes for 2 years; and (2) determine changes in the metabolomic profile over the training period. The starting hypotheses were that the metabolic profile of horses differs between training programmes and that it changes substantially over time. Targeted metabolomics was performed and a hypothesis-generating approach was applied. To our knowledge, this is the first absolute quantitative metabolomics study in race horses subjected to a long-term exercise programme (2 years).

## Material and methods

The study was performed at the Swedish National Centre for Trotting Education at Wången. The protocol was approved by the Ethics Committee, Umeå, Sweden (D.nr:A90-10, 2010-09-14) and all experiments were performed in accordance with relevant guidelines and regulations. The reporting of the experiments are in accordance with the ARRIVE guideline.

### Horses and management

Sixteen Standardbred 1.5-year-old male horses from four breeders were included in the study. They entered the study in September 2010 and it ended in December 2012. All horses were castrated in December 2010 or January 2011. They were housed in individual boxes for about 16 h a day from Monday to Thursday/Friday, while for the rest of the time they were kept in a paddock with shelter. Their diet consisted of haylage with known energy and nutrient content and both haylage and water were available ad libitum in the paddock and the box. The diet was supplemented with pelleted lucerne (Krafft AB, Malmö, Sweden), a vitamin and mineral supplement (Krafft AB, Malmö, Sweden) and NaCl to meet the nutrient requirements of horses of this age and training intensity^7^. Daily intake of metabolisable energy was 123 ± 16 MJ/100 kg body weight (BW) and daily intake of crude protein was decreased over time as the requirement for growth decreased (428–354, 373–321 and 321–234 g/100 kg BW as 1-, 2- and 3-year-olds, respectively). Results of all nutrient analyses of haylage and more data on feed, energy and nutritional intake can be found in previous publications^8,9^. Body condition was scored every other month and was 4.8–5.1 (9-point scale) throughout the study^9^.

### Training

The training programmes were planned together with three professional trainers and was based on common practise with respect to training methods and age of the horses. From autumn as 1.5-year-olds to spring as 2-year-olds, all horses were exposed to the same training programme, which began with breaking in the autumn and progressed to trotting with a jog cart 4 times/week. Speed was gradually increased to 5.6 m/s and distance to 5–7 km. In the middle of March as 2-year-olds, high-intensity training (i.e. heart rate > 180 bpm, Polar CS600X, Polar Electro, Finland) was introduced and the horses were divided into a High training group or a Low training group. These groups were balanced with respect to breeder and parameters known to affect performance, such as age in days, genetic potential (sire and mean pedigree index estimated with the Best Linear Unbiased Prediction (BLUP) method), percentage of French ancestry, inbreeding coefficient, height at withers, abnormal radiographic findings, conformation and proportion of type IIA/type IIB muscle fibres^10^. The High group underwent a training programme designed by a group of professional trainers that consisted of two high-intensity training sessions per week involving heat training, interval training or uphill interval training (Table 1), and 1–2 jogging sessions. The Low group had the same training programme as the High group, but with 30% lower high-intensity training distance. For example, during interval training horses in the High group performed six repetitions and horses in the Low group performed only four repetitions (Table 1). Similar velocity was aimed for with both training groups. More information about actual training distances and training sessions can be found in a previous publication^10^. All horses were subjected to exercise tests and an improvement in the cardiovascular response was observed as well as in the lactate threshold during the summer as 3-year-olds^8,10^.

**Table 1:** Weekly high-intensity training (heart rate > 180 bpm) sessions in Standardbred horses divided into two training groups (high and low).

Training type	High group (n = 8)		Low group (n = 8)	
	2 years old	3 years old	2 years old	3 years old
Heat	1–2*1600 m	2–3*1600 m	1–2*1100 m	2–3*1100 m
Interval	6*500–700 m	6*700 m	4*500–700 m	4*700 m
Uphill interval		6*600 m		4*600 m

### Blood sample collection

Blood samples were collected on four different occasions between 2010 and 2012 (December as 1.5-year-olds, July as 2-year-olds (14 weeks after high-intensity training was introduced), December as 2.5-year-olds, and December as 3.5-year-olds). All samples were collected in the morning (05.00–06.00 h, minimum 15 h after a training session) in each horse’s box, using the vacutainer technique with blood extracted into lithium heparin tubes (10 mL) from the jugular vein. Immediately after collection, all blood samples were centrifuged at room temperature (10 min, 2700 rpm, 920×*g*) and then the plasma was frozen at − 20 °C for later analyses.

### Metabolomics analysis using mass spectrometry

The samples were analysed at the Mass Spectrometry Based Metabolomics Facility in Uppsala, Sweden, in 2021. The MxP® Quant 500 kit (Biocrates, Innsbruck, Austria) was used for targeted measurement of different classes of metabolites, following the manufacturer’s recommended protocols. This kit enables targeted absolute quantitative analysis of up to 630 metabolites, including amino acids (AA), biogenic amines, acylcarnitines, glycerophospholipids, sphingolipids and sum of hexoses, allowing for comprehensive assessment of a broad range of analytes and metabolic pathways in a single targeted assay. Sample analysis involved a combination of tandem mass spectrometry (MS/MS), flow-injection analysis (FIA) and ultra-high performance liquid chromatography (UPLC). To ensure accurate quantification, chemically homogenised and isotope-labelled internal standard mixture (IS) was used. Sample preparation began with addition of 10 µL horse plasma to each well on a 96-well plate containing IS dried under a nitrogen stream using a positive pressure manifold (Biostage, Uppsala, Sweden), together with 50 µL 5% phenyl isothiocyanate solution. The plate was incubated at room temperature for 1 h and then dried again. To extract the metabolites, 300 µL 5 mM ammonium acetate in methanol were pipetted onto a filter and incubated for 30 min. The extract was eluted into a new 96-well plate using positive pressure. For further UPLC-MS/MS analyses, 150 µL extract were diluted with an equal volume of ultra-pure water. For FIA-MS/MS analyses, 10 µL extract were diluted with 490 µL FIA solvent (provided by Biocrates). After dilution, UPLC-MS/MS and FIA-MS/MS measurements were performed. For chromatographic separation, an UPLC system coupled to a TQS mass spectrometer in electrospray ionisation (ESI) mode was used. Data were recorded using the Analyst Mass Link software and transferred to the MetIDQ software (version Oxygen-DB110-3005), which was used for further data processing. All metabolites were identified using isotopically labelled internal standards and by multiple reaction monitoring (MRM) using optimised MS conditions as provided by Biocrates. Concentrations of metabolites were quantified using a seven-point calibration curve, depending on the metabolite class. The metabolites were used to calculate different sums and ratios and are listed in Supplementary Material SM1 and SM2. The total plasma protein concentration was analysed in all samples prior to the metabolomics analysis, in order to ensure that freeze drying had not occurred during storage.

### Statistical analyses

Metabolites, sums and ratios of metabolites which were below the limit of detection (LOD) for all horses and time points were excluded from the analysis. For metabolites which were below LOD for one time point but were detectable at another time point, a value corresponding to 10% of the LOD cut-off value was used. Sums and ratios of metabolites which had fewer than four values above LOD per training group (in analyses of training groups) or per age (in analyses of change over time) were removed. In analyses of training group, a linear model which included the effect of training group was used and the four different ages were run separately. In analyses of changes over time, a linear model which included the effect of training group and age was used and the sampling ages (2, 2.5 and 3.5 years) were compared with the age at which horses entered the study (1.5 years). A total of 624 metabolites and 196 sums and ratios (n = 820) were analysed. All statistical analyses were performed in R (v4.1.2, R Core Team, 2022). Differences were considered significant at *p* < 0.05. All values were log-transformed. The *p* values were adjusted using the false recovery rate (FDR) method^11^, where all *p* values were ranked in ascending order and assigned a ranking number, and then the adjusted *p* value was calculated as: *p* value x (Total number of tests/Ranking number). A volcano plot was made by plotting − log_10_(Adjusted *p* value) on the y-axis and log_2_(Fold change) on the x-axis. Fold change was calculated for each metabolite by dividing mean value for the High group by mean value for the Low group. All *p* values presented below are adjusted *p* values unless otherwise stated.

## Results

### Differences between training groups

**Figure 1:**
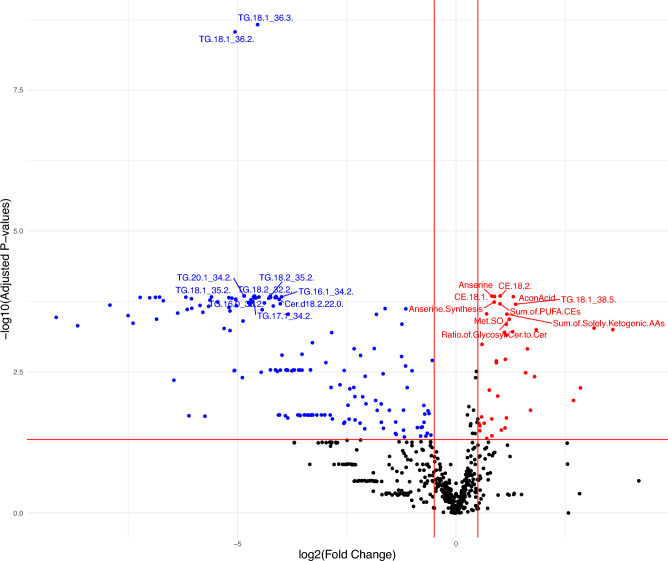
Volcano plot of plasma metabolites present in higher (red), lower (blue) and unchanged (black) concentrations in plasma samples from the High group compared with the Low group of 16 Standardbred horses at 2 years of age. Upper left quadrant contains adjusted *p* values < 0.05 and fold change < − 0.5, upper right quadrant adjusted *p* values < 0.05 and fold change > 0.5. Abbreviations: aconitic acid (AconAcid), cholesteryl ester (CE), methionine-sulfoxide (Met.SO), ceramides (Cer), polyunsaturated fatty acids (PUFA), amino acids (AA), triglyceride (TG).

Quantitative MS analyses of the 820 metabolites, ratios and sums of metabolites revealed significant differences between the training groups when the horses were 2 years old, but not at other ages (Table 2). At 2 years of age, 161 metabolites had lower values and 51 had higher values in the High group compared with the Low group (Fig. 1). Quantitative measures for all metabolites, ratios and sums of metabolites included in statistical analyses can be found in SM1.

**Table 2:** Number of metabolites and sums and ratios of metabolites included in statistical analyses for the different sampling ages, and number that were significantly different (FDR-adjusted *p* < 0.05) at different ages.

Age	No. analysed	No. of metabolites included in statistical analyses			
		1.5 years old	2 years old	2.5 years old	3.5 years old
Metabolites	624	532	532	532	532
Sums and ratios	196	136	128	134	134
FDR-adjusted *p* values < 0.05		0	212	0	0

The 10 most significant metabolites with higher concentrations in the High group compared with the Low group included two metabolites related to AA, two cholesteryl esters (CEs), one carboxylic acid, one triglyceride (TG), and four sums and ratios. The 10 most significant metabolites with lower values in the High group compared with the Low group comprised of one ceramide (Cer) and nine TG metabolites (Table 3).

**Table 3:** Plasma metabolites with the most significant FDR-adjusted *p* values for the difference in mean values for 16 2-year-old Standardbred horses divided into two different training groups (High and Low).

Higher in high group		Lower in high group	
Metabolite	FDR *p* value	Metabolite	FDR *p* value
Aconitic acid	1.5e−04	Cer.d18.2.22.0	1.5e−04
Anserine	1.4e−04	TG.16.0_38.2	1.4e−04
Anserine Synthesis	1.8e−04	TG.16.1_34.2	1.5e−04
CE.18.1	1.5e−04	TG.17.1_34.2	1.5e−04
CE.18.2	1.4e−04	TG.18.1_35.2	1.5e−04
Methionine-Sulfoxide	3.0e−04	TG.18.1_36.2	3.0e−09
Ratio of Glycosyl Cer to Cer	3.7e−04	TG.18.1_36.3	2.2e−09
Sum of PUFA CEs	2.0e−04	TG.18.2_32.2	1.4e−04
Sum of Solely Ketogenic AAs	3.0e−04	TG.18.2_35.2	1.4e−04
TG.18.1_38.5	2.0e−04	TG.20.1_34.2	1.4e−04

*CE* Cholesteryl ester, *Cer* ceramides, *PUFA* polyunsaturated fatty acids, *AA* amino acids, *TG* triglyceride.

### Differences between time points

**Figure 2:**
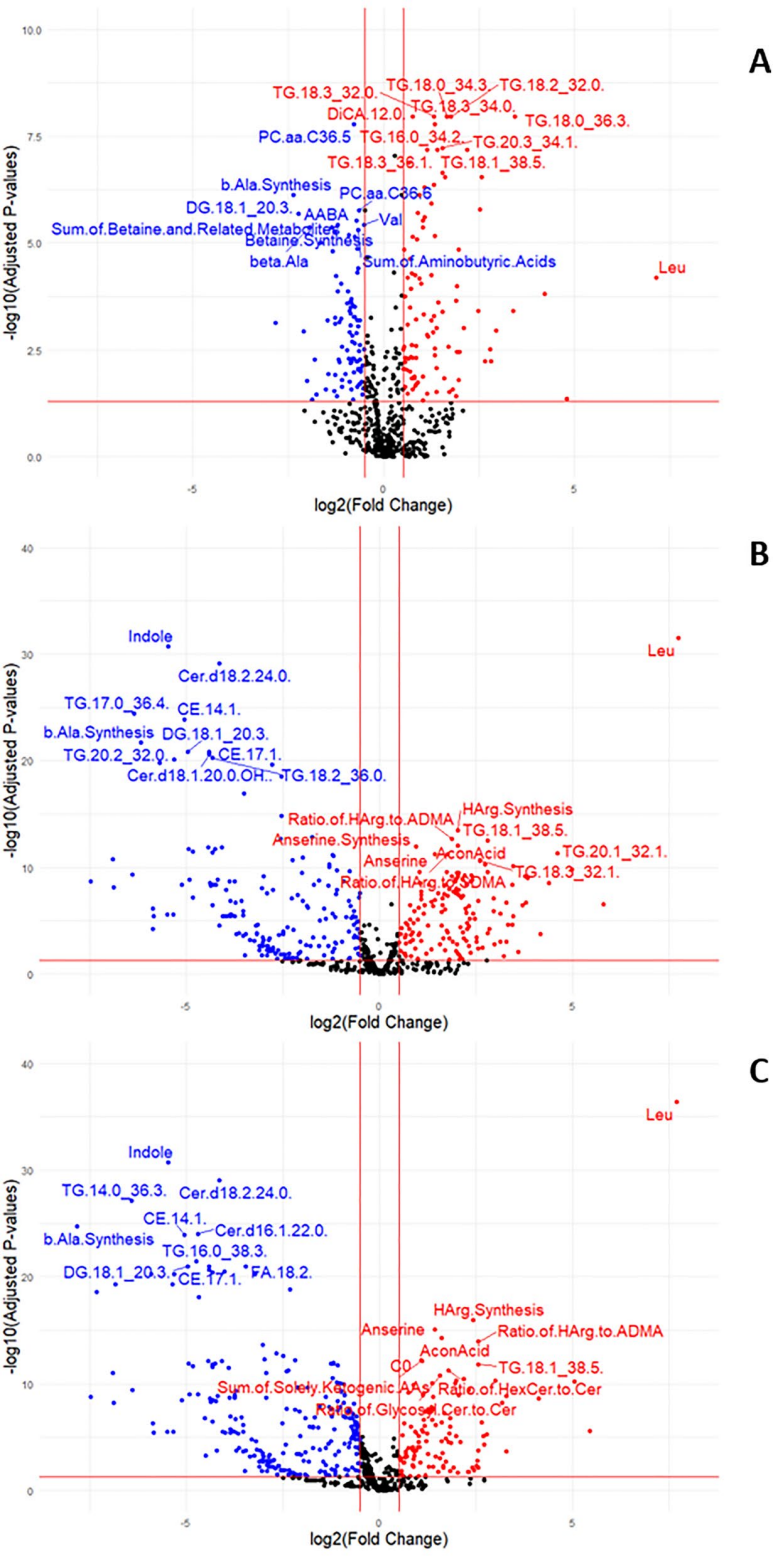
Volcano plot of plasma metabolites with Higher (red), lower (blue) and unchanged (black) values at (**A**) 2 years of age, (**B**) 2.5 years of age and (**C**) 3.5 years of age compared with 1.5 years of age for 16 Standardbred horses in training. Upper left quadrant contains adjusted *p* values < 0.05 and fold change < − 0.5, upper right quadrant adjusted *p* values < 0.05 and fold change > 0.5. Note different scale on the y-axis in panel A (0–10) compared with B and C (0–40). *AconAcid* aconitic acid, *AA* amino acids, *AABA* alpha-aminobutyric acid, *b.Ala* beta alanine, *C0* carnitine, *Cer* ceramides, *CE* cholesteryl ester, *DG* diglyceride, *ADMA* asymmetric dimethylarginine, *DiCA* dodecanedioic acid, *FA* fatty acid, *HexCer* hexosylceramides, *HArg* homoarginine, *Leu* leucine, *PC* phosphatidylcholines, *SDMA* symmetric dimethylarginine, *TG* triglyceride, *Val* valine.

Compared with age 1.5 years (before the high-intensity training started), multiple metabolites differed significantly in concentration at 2, 2.5 and 3.5 years of age (Table 4). At 2 years of age, 133 metabolites had higher values and 129 had lower values than at 1.5 years of age (Fig. 2A). At 2.5 years of age, 176 metabolites had higher values and 218 had lower values than at 1.5 years of age (Fig. 2B). At 3.5 years of age, 143 metabolites had higher values and 314 had lower values than at 1.5 years of age (Fig. 2C). Quantitative measures for all metabolites, ratios and sums of metabolites included in the statistical analyses can be found in SM2.

**Table 4:** Number of metabolites and sums and ratios of metabolites included in statistical analyses of the different sampling ages (2, 2.5 and 3.5 years) compared with 1.5 years of age, and number that were significantly different (FDR-adjusted *p* < 0.05) at different ages.

Age	No. analysed	No. of metabolites included in statistical analyses		
		2 years old	2.5 years old	3.5 years old
Metabolites	624	524	510	509
Sums and ratios	196	137	132	133
FDR-adjusted *p* values < 0.05		262	394	457

The 10 most significant metabolites with higher concentrations at 2, 2.5 and 3.5 years of age compared with 1.5 years of age are shown in Table 5. At 2 years of age, nine TG metabolites and one carboxylic acid showed higher values than at 1.5 years of age. At 2.5 years of age, one AA, one AA-related compound, one carboxylic acid, three TG metabolites and four sums and ratios of metabolites had higher values than at 1.5 years of age. At 3.5 years of age, one AA, one AA-related compound, one carboxylic acid, one TG metabolite, one carnitine and five sums and ratios of metabolites had higher values than at 1.5 years of age.

**Table 5:** Plasma metabolites found in significantly higher concentrations (most significant FDR-adjusted *p* values for the difference between means) in 16 Standardbred horses in training at ages 2, 2.5 and 3.5 years compared with 1.5 years of age.

2 years of age		2.5 years of age		3.5 years of age	
Metabolite	FDR *p* value	Metabolite	FDR *p* value	Metabolite	FDR *p* value
DiCA.12.0	1.1e−08	Aconitic acid	9.4e−13	Aconitic acid	4.8e−15
TG.16.0_34.2	1.7e−08	Anserine	5.2e−12	Anserine	8.6e−16
TG.18.0_34.3	1.1e−08	Anserine synthesis	1.1e−12	Carnitine	6.0e−13
TG.18.0_36.3	1.1e−08	HArg synthesis	3.5e−14	HArg synthesis	1.2e−16
TG.18.1_38.5	6.6e−08	Leucine	2.9e−32	Leucine	4.0e−37
TG.18.2_32.0	1.1e−08	Ratio of HArg to ADMA	1.9e−13	Ratio of glycosyl Cer to Cer	1.9e−11
TG.18.3_32.0	1.1e−08	Ratio of HArg to SDMA	5.4e−12	Ratio of HArg to ADMA	1.2e−14
TG.18.3_34.0	1.1e−08	TG.18.1_38.5	3.0e−13	Ratio of HexCer to Cer	6.0e−12
TG.18.3_36.1	6.6e−08	TG.18.3_32.1	2.2e−11	Sum of solely ketogenic AAs	6.9e−13
TG.20.3_34.1	5.9e−08	TG.20.1_32.1	4.9e−12	TG.18.1_38.5	1.5e−12

*AA* amino acids, *ADMA* asymmetric dimethylarginine, *DiCA* dodecanedioic acid, *HexCer* hexosylceramides, *HArg* homoarginine, *Cer* ceramides, *SDMA* symmetric dimethylarginine, *TG* triglyceride.

The 10 most significant metabolites with lower concentrations at 2, 2.5 and 3.5 years of age compared with 1.5 years of age are shown in Table 6. At 2 years of age, one carboxylic acid, one biogenic amine, one diglyceride (DG), two phosphatidylcholines (PC), one AA and four sums and ratios of metabolites showed lower values than at 1.5 years of age. At 2.5 years of age, two CE, two Cer, one DG, one indole, three TG and one ratio of metabolites had lower values than at 1.5 years of age. At 3.5 years of age, two CE, two Cer, one DG, one indole, one ratio of metabolites, two TG and one fatty acid (FA) had lower values than at 1.5 years of age.

**Table 6:** Plasma metabolites found in significantly lower concentrations (most significant FDR-adjusted *p* values for the difference between means) in 16 Standardbred horses in training at 2, 2.5 and 3.5 years of age compared with 1.5 years of age.

2 years of age		2.5 years of age		3.5 years of age	
Metabolite	FDR *p* value	Metabolite	FDR *p* value	Metabolite	FDR *p* value
Alpha-aminobutyric acid	3.0e−06	Beta alanine synthesis	1.8e−22	Beta alanine synthesis	1.9e−25
Beta alanine synthesis	7.5e−07	CE.14.1	1.5e−24	CE.14.1	1.1e−24
Beta alanine	4.3e−06	CE.17.1	1.4e−21	CE.17.1	9.5e−22
Betaine synthesis	4.4e−06	Cer.d18.1.20.0.OH	2.7e−21	Cer.d16.1.22.0	9.3e−25
DG.18.1_20.3	2.1e−06	Cer.d18.2.24.0	7.9e−30	Cer.d18.2.24.0	8.3e−30
PC.aa.C36.5	1.7e−08	DG.18.1_20.3	1.4e−21	DG.18.1_20.3	9.5e−22
PC.aa.C36.6	1.7e−06	Indole	1.8e−31	FA.18.2	9.5e−22
Sum of aminobutyric acids	4.9e−06	TG.17.0_36.4	4.1e−25	Indole	1.8e−31
Sum of betaine and rel. metab	3.7e−06	TG.18.2_36.0	5.5e−21	TG.14.0_36.3	7.5e−28
Valine	3.7e−06	TG.20.2_32.0	7.7e−21	TG.16.0_38.3	3.6e−22

*Cer* ceramides, *CE* cholesteryl ester, *DG* diglyceride, *FA* fatty acid, *PC* phosphatidylcholines, *TG* triglyceride.

## Discussion

This study evaluated the metabolic profile in horses subjected to two different long-term training programmes until fit to race as 3.5-year-olds. To our knowledge, this is the first long-term evaluation of the metabolic profile in young harness horses exposed to long-term training.

Comparison of the two training groups (High and Low) revealed significant differences in metabolic profile at 2 years of age. This was approximately 3.5 months after the horses had started high-intensity training, in which the Low group was subjected to 30% shorter distances compared with the High group, and it coincided with observations of significant differences in cardiovascular responses between training groups^10^. At 2 years of age, leucine and solely ketogenic AA (leucine plus lysine) levels were significantly higher in the High group compared with the Low group (SM1) and significantly higher over time at all ages compared with 1.5 years of age (SM2). This is in agreement with pre-exercise results from a 12-week high-intensity training study by Klein et al.^4^ However, in another study with 18 weeks of high to moderate training intensity, no effect on plasma leucine levels at rest was observed^12^. This difference may be due to different training intensities or to high training intensity being required for this change to be manifested. Branched-chain amino acids (BCAA) are involved in aerobic energy metabolism in skeletal muscle although only as a minor contributor, i.e. about 1–15% during exercise^13^. It has also been shown that leucine stimulates protein synthesis in skeletal muscles by hyperphosphorylating proteins involved in mRNA translation, which leads to both an increase in activity and synthesis of these proteins^14^. It is also possible that leucine stimulates glycogen synthesis^15^, which is of relevance for tissue development and performance^16^. The results in the present study support the claim that leucine is an important metabolite for exercise performance, since it was altered depending on training group and also over time.

Anserine and anserine synthesis (anserine divided by carnosine) levels were significantly higher in the High group compared with the Low group. The levels of both also increased significantly over time (SM2), indicating an effect of both intensity and training period. Anserine is a methylated variant of carnosine and in horses anserine and carnosine can be found in neuronal tissue and skeletal and heart muscle^17^. It is believed that carnosine and anserine play a major role in maintaining intracellular buffering and pH-balance^17,18^, but it may also have anti-oxidant, anti-glycation and anti-lipoxidation functions^17^. All of these activities could be of relevance for exercise performance. There is an obvious need for pH-buffering in the exercising Standardbred horse, because during high-intensity exercise lactic acid is produced by the skeletal muscle, resulting in high muscle and plasma lactate levels (> 70 mmol/kg and > 30 mmol/L, respectively^19^), as well as lowered plasma pH during exercise at the lactate threshold^20^. The importance of lactate and proton removal from muscle has been studied^21^ and in Standardbred horses efficient (monocarboxylate) transporters have been identified in both muscle and red blood cells^22^. While transport of lactate into red blood cells is a passive means (sink) to increase the muscle-plasma concentration gradient, oxidation of lactate in the liver and inactive muscles is more efficient^22^. Lactate is oxidized to pyruvate, which can be metabolized through the tricarboxylic acid cycle or converted to glucose through gluconeogenesis. The role of lactate in the delivery of oxidative and gluconeogenic substrates and for cellular signalling (the lactate shuttle concept^23^) is probably of major importance for the development and performance of race horses. Several of the changes in aerobic energy metabolism detected could in principle be triggered by lactate, but that needs further investigation. There was no significant difference in carnosine itself in this study, but there was a significant decrease in beta alanine and beta alanine synthesis (beta alanine divided by carnosine) levels in the High group (SM1) and over time (SM2). Beta alanine is the rate-limiting factor for synthesis of carnosine within muscle, and in humans beta alanine supplementation has positive effects on exercise performance in which acidosis contributes to fatigue^24^. These results suggest that the duration of high-intensity training in each training session may be important for development of pH-buffering capacity.

Another AA involved in reducing oxidative stress is methionine^25^, which is easily oxidised to methionine sulfoxide. The concentration of this metabolite was higher in the High group than in the Low group. However, there was no consistent change over time, with both an increase (2 years old) and a decrease (3.5 years old) compared with 1.5 years of age (SM2).

The levels of several triglyceride metabolites significantly decreased in the High group compared with the Low group. However, over time there was an increase in the levels of most triglyceride metabolites, which is in agreement with findings in a longitudinal study of horses in a 12-week training programme^4^. This could be because fat is an important source of energy during exercise in Standardbred horses. It is known that the activity of 3-hydroxyacyl-CoA-dehydrogenase, the last step in beta-oxidation, may increase with training in Standardbred horses^26^ and also that it is associated with increased performance^27^. Our results also showed that aconitic acid levels were higher in the High group compared with the Low group, and were higher at 2.5 and 3.5 years of age compared with 1.5 years of age. Aconitic acid is an intermediate in the citric cycle. It is well known that training increases the activity of citrate synthase (catalyses the first reaction in the citric cycle) in the gluteus muscle of horses^26–29^ and our results indicate increased activity of the citric cycle. Altogether, our findings support the suggestion by Klein et al.^4^ that metabolites related to amino acid and lipid metabolism play pivotal roles in the response of equine skeletal muscle to training.

The levels of ceramide metabolites were significantly lower in the High group compared with the Low group, and also decreased over time compared with 1.5 years of age. There are few previous studies on the effects of ceramide levels in response to exercise, but our finding of a decrease over time is in agreement with observations in obese humans undergoing a 12-week training programme with high to moderate training intensity^30^. Ceramides are bioactive molecules important in e.g. inflammatory processes and apoptosis, and high levels have been linked to insulin resistance^31^. In horses, it has been suggested that high plasma ceramide concentrations indicate insulin dysregulation^32^. Exercise and training can improve insulin resistance in young humans^33^ and there is evidence of a similar response in horses^34,35^, although conflicting observations have been reported^36^.

In the High group, HArg and HArg synthesis (HArg divided by the sum of arginine and lysine) levels increased compared with the Low group (SM1) and also increased over time at all ages compared with 1.5 years of age. The ratio of HArg to asymmetric dimethylarginine (ADMA) was also higher in the High group compared with the Low group (SM2) and both the ratio of HArg to ADMA and the ratio of HArg to symmetric dimethylarginine (SDMA) were higher for all ages compared with 1.5 years of age. HArg is suggested to be one of the substrates for nitric oxide (NO) synthesis and ADMA as an inhibitor of NO synthesis, but little is known about this as yet^37,38^. Nitric oxide is one of the smallest signalling molecules and has several biological functions, including as a potent vasodilator^38^. There is evidence that physical activity enhances NO production and also that NO improves performance and promotes recovery^39^. An altered vascular response is in line with the significant changes (improvements) in cardiovascular responses in the High group, by 2.5 years of age and throughout the rest of the study, which we reported previously in these horses^10^.

Indole is produced when tryptophan is metabolised in the gut by bacterial tryptophanases and is then absorbed to the circulation^40^. Indole is suggested to have beneficial effects on human health by influencing e.g. oxidative stresses and intestinal inflammation^41^. However, it appears to have dual effects on the host^42^. For example, indole seems to have beneficial effects on the intestinal mucosa, but high levels have negative impacts on emotional behaviour and anxiety^42,43^. In the present study, indole concentrations were not affected by training group, but the concentrations were lower at age 2.5 and 3.5 years compared with 1.5 years of age. This contradicts findings for humans, where increases in microbiome-derived tryptophan metabolites has been reported in soldiers subjected to an 80-day training programme^44^. The reason for this discrepancy in results is unclear, but could be due to diet and changes in body composition i.e. weight loss in the study on humans^45^ and growth but maintained body fat content in our study^9^. Another explanation is simply that the reduction in our study was due to the decrease in crude protein intake (and thereby tryptophan), as the crude protein requirement for growth decreased over time^9^.

Cholesteryl ester is an esterified variant of cholesterol which is either stored in the cell or released into the blood^46^. Cholesterol is involved in cell membrane structure and function, and also in binding numerous transmembrane proteins^46^. It is difficult to draw any firm conclusions about cholesteryl esters based on the results in the present study, because there were both significant increases and decreases in the High group compared with the Low group (SM1) and the same was true for changes over time (SM2).

In this study, differences between the training groups were only observed at 2 years of age, although the different training programmes were followed throughout the study until age 3.5 years. A likely explanation, and a limitation of this study, is that strict compliance with the training programme was not possible during the latter part of the study, especially in the High group, due to variations in the fit-to-train status. Horses which were not fit to train according to the trainer were allowed to skip a training session and this resulted in horses in the High group missing more training than horses in the Low group. From July as 2-year-olds until June as 3-year-olds the Low group performed 4–5 more training sessions per 3-month period compared to the High group and during the last 6 months of the study, the Low group had _~_2 more sessions per 3-month period^10^. Consequently, from the age of 2.5 years the horses in the High training group received on average the same volume of training as horses in the Low group^47^. Still, each training session performed by the High group had longer duration/distance than in the Low group and differences in the metabolic adaption could therefore potentially be expected. However, knowledge of the importance of variations in frequency and duration of training sessions is limited in horses. As mentioned earlier, this study is unique in the exploration of the effect of different training programs for young horses over a period relevant to true race training conditions.

Another limitation of this study is the lack of a non-trained control group, i.e. a group subjected to no training at all. The main aim of the study was to explore differences in the metabolomic profile in response to two different training programmes, where the High group could be considered the control treatment (comparable to standard practise^9^). In the absence of significant differences between training programmes during the latter part of the study, a design with a third, non-exercised control group would have been of great academic interest. This was part of the initial experimental plans but turned out to be a too complex and expensive design. Accordingly, for several metabolites we observed significant differences over time, but were unable to discriminate potential training effects from effects caused by changes related to age or growth. However, a study by Roneus et al.^29^ showed that training, not growth, is the main factor inducing high oxidative capacity in muscle of Standardbred horses in training. Another potential limitation in this study is that the samples analysed had been frozen for 9–11 years, which may have affected the results if metabolites oxidise or water evaporates. However, the levels observed for numerous metabolites (e.g. AAs and triglycerides) were within the range reported previously for horses at rest^12,48,49^ (our horses had been at rest for at least 15 h when blood samples were collected), which implies that these metabolites were well preserved during storage.

The strength of this study is the selected study population of elite performance horses training for competition under ‘real world’ conditions. Information about the metabolic profile of the individuals included in the present study can be used for future studies on profiles of importance for athletic performance and longevity. We have earlier shown that the proportion of experimental horses that raced as 3-year-olds was equal to that of their siblings, and greater than the proportion of cohort geldings^50^. We also know that four horses were not active after the 3-year-old season and that the rest were fit to race until at least 8 ± 2 years of age.

In conclusion, the training programmes applied in the present study promoted physiological adaptations linked to aerobic energy production, induced changes in amino acid metabolism and potentially also affected pH-buffering and vascular and insulin responses. An increase in the distance covered in high-intensity exercise seemed to stimulate these changes even more. Further studies are needed to link the metabolic profile of young horses in training to performance and longevity.

### Supplementary Information

Supplementary Information.

## Data Availability

The datasets used and analysed during the current study are available from the corresponding author on reasonable request.
